# Compound K Attenuates the Development of Atherosclerosis in ApoE^−/−^ Mice via LXRα Activation

**DOI:** 10.3390/ijms17071054

**Published:** 2016-07-08

**Authors:** Li Zhou, Yu Zheng, Zhuoying Li, Lingxia Bao, Yin Dou, Yuan Tang, Jianxiang Zhang, Jianzhi Zhou, Ya Liu, Yi Jia, Xiaohui Li

**Affiliations:** 1 Institute of Materia Medica and Department of Pharmaceutics, College of Pharmacy, Third Military Medical University, Shapingba, Chongqing 400038, China; zl1007@tmmu.edu.cn (L.Z.); zhengyu@tmmu.edu.cn (Y.Z.); linn0626@163.com (L.B.); douyin@tmmu.edu.cn (Y.D.); tangyuan@tmmu.edu.cn (Y.T.); jxz@tmmu.edu.cn (J.Z.); jazi@tmmu.edu.cn (J.Z.); liuya@tmmu.edu.cn (Y.L.); 2 Department of pharmacy, Xinqiao Hospital & The Second Affiliated Hospital, Third Military Medical University, Shapingba, Chongqing 400037, China; 3 Department of Outpatient, Logistical Engineering University of PLA, Shapingba, Chongqing 401311, China; jaiyi1979@163.com

**Keywords:** atherosclerosis, compound K, reverse cholesterol transport, inflammasome, LXRα

## Abstract

Background: Atherosclerosis is a fundamental pathological process responded to some serious cardiovascular events. Although the cholesterol-lowering drugs are widely prescribed for atherosclerosis therapy, it is still the leading cause of death in the developed world. Here we measured the effects of compound K in atherosclerosis formation and investigated the probably mechanisms of the anti-antherosclerosis roles of compound K. Methods: We treated the atherosclerotic model animals (apoE^−/−^ mice on western diet) with compound K and measured the size of atherosclerotic lesions, inflammatory cytokine levels and serum lipid profile. Peritoneal macrophages were collected in vitro for the foam cell and inflammasome experiments. Results: Our results show that treatment with compound K dose-dependently attenuates the formation of atherosclerotic plaques by 55% through activation of reverse cholesterol transport pathway, reduction of systemic inflammatory cytokines and inhibition of local inflammasome activity. Compound K increases the cholesterol efflux of macrophage-derived foam cells, and reduces the inflammasome activity in cholesterol crystal stimulated macrophages. The activation of LXRα may contribute to the athero-protective effects of compound K. Conclusion: These observations provide evidence for an athero-protective effect of compound K via LXRα activation, and support its further evaluation as a potential effective modulator for the prevention and treatment of atherosclerosis.

## 1. Introduction

Atherosclerosis is a fundamental pathological process that works in response to some serious cardiovascular diseases, including stroke and coronary artery disease. Despite the efficacious lipid-lowering treatments, such as atorvastatin, used in therapy, atherosclerosis disease remains the leading cause of death in developing nations, accounting for one-fifth of all deaths in the world [1]. Besides hyperlipidemia, NLRP3 inflammasome activation in aorta is considered to play a critical role in the formation of atherosclerosis lesions. Hence, there is a need for further development of more effective therapeutic approaches.

The root of *Panax notoginseng* (Burk.) F. H. Chen has been used as a health product and natural remedy in traditional medicine for cardiovascular diseases for more than 1000 years in Asia, including China, Japan and Korea. Our previous studies showed that *Panax notoginseng* saponins, the major effective ingredients extracted from *Panax notoginseng*, exhibited noticeable anti-atherogenic effects via blood lipid profiles regulation and anti-inflammatory capability [2]. However, the exact molecular mechanisms of *Panax notoginseng* saponins are hard to investigate, because it contains a huge number of components. Studies showed that only compound K (20-O-b-d-glucopyranosyl-20(S)-protopanaxadiol) was found in blood of rats after oral administration of intact Rb1 [3], the major component of *Panax notoginseng* saponins (>30%). This indicated that compound K might be one of the active metabolites of *Panax notoginseng* saponins. It has been reported that compound K has multiple biological activities, including inhibition of the proliferation of myeloid leukemia, pulmonary adenocarcinoma, gastric adenocarcinoma and hepatoma cell lines [4,5,6], activation of glucocorticoid receptor [7], and so on. Our recent study revealed that the role *Panax notoginseng* saponins plays in reducing the formation of foam cells is associated with increased level of ABCA1 (ATP-binding cassette transporter A1) [8], a major down-stream product of LXRα. In addition, *Panax notoginseng* saponins can attenuate atherosclerosis lesions in rats by induction of LXRα expression [9]. Because studies have shown that synthetic LXR agonist, such as GW3965 and T090317, can significantly attenuate the formation of atherosclerosis lesions via promotion of reverse cholesterol transpot (RCT), we suppose that compound K might play anti-atherosclerosis roles via LXRα activation. In addition, compound K can reduce fatty acid-induced lipid droplets deposition in hepatocytes in vitro [10], the major side effect of synthetic LXRα agonists. Therefore, compound K might have potential to be used in atherosclerosis therapy.

In the present study, we investigated the effects of compound K on NLRP3 inflammasome activation and RCT promotion in C57BL/6 apoE^−/−^ mice on western diet. Then we tried to explore the probably mechanisms of the athero-protection effects of compound K.

## 2. Results

### 2.1. Compound K Attenuates Atherosclerotic Lesion and Fatty Liver in ApoE^−/−^ Mice

**Figure 1:**
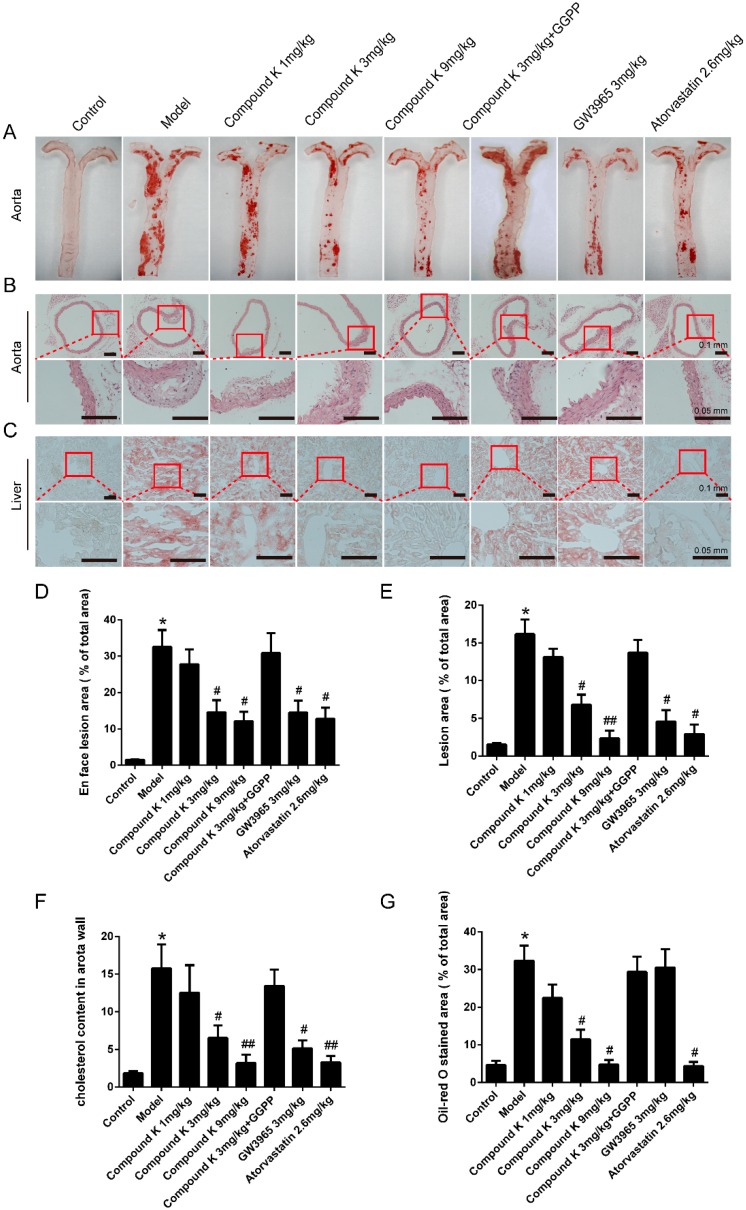
Compound K attenuates atherosclerosis lesion formation and fatty liver in apoE^−/−^ mice. *En face* (**A**) and aortic sinus section (**B**), (zoomed areas were marked with red square) images of atherosclerosis were analysed by NIS-Elements image analysis software, and the cartograms were shown as (**D**,**E**). Levels of cholesterol concentration in mice aorta were extracted and measured (**F**). Levels of lipid deposition in apoE^−/−^ mice livers were measured using Oil-Red O staining (**C**). Images of stained liver histological sections were analysed by NIS-Elements image analysis software (**G**). Data were presented as mean ± SEM (*n* = 6) and analysed by ANOVA with Dunnett’s post-hoc analysis. * *p* < 0.05 vs. control; # *p* < 0.05 vs. model; ## *p* < 0.01 vs. model.

As shown in Figure 1, mice treated with compound K (3 and 9 mg/kg) showed a significant decrease in en face atherosclerotic lesions compared with the model group by 55% and 63% (Figure 1A,D), consistent with the data of aortic sinus sections (Figure 1B,E) and cholesterol content of the aorta wall (Figure 1F). Similar changes were observed in GW3965 and atorvastatin groups.

Abundant oil-red-O-stained lipid depositions were observed in mice livers of model group, GGPP group and GW3965 group, and the relative values of positive stained area were 32.34% ± 4.05%, 29.39% ± 4.07% and 30.57% ± 4.86%, respectively. Significant decrease of the percentage was found in compound K 3 mg/kg (11.85% ± 2.40%) and 9 mg/kg (4.07% ± 0.76%) groups, as well as in atorvastatin (4.39% ± 1.08%) group (Figure 1C,G).

### 2.2. Compound K Decreases Serous Inflammatory Cytokines and Modulated Serum Lipid Profiles in ApoE^−/−^ Mice

**Figure 2:**
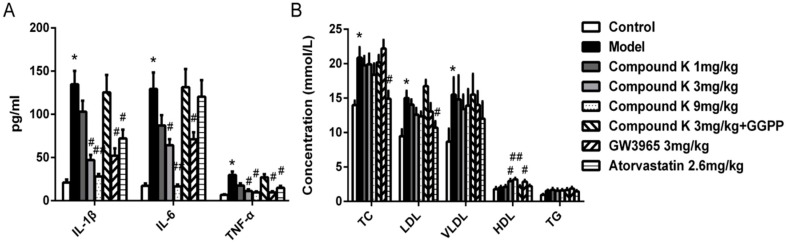
Effects of compound K on serous lipid profile and inflammatory cytokines in apoE^−/−^ mice. Serous total cholesterol (TC), low density lipoprotein cholesterol (LDL), very low density lipoprotein cholesterol (VLDL), high density lipoprotein cholesterol (HDL) concentrations and triglyceride (TG) in apoE^−/−^ mice treated with different concentrations of compound K were measured using Olympus AU-2700 automatic biochemical analyzer (**A**); Levels of serum IL-1β, IL-6 and TNF-α were detected by Mcytomag-70K-3 Mouse Cytokine/Chemokine Magnetic Bead Panel (**B**). Data are presented as mean ± SEM (*n* = 6) and analysed by ANOVA with Dunnett’s post-hoc analysis. * *p* < 0.05 vs. control; # *p* < 0.05 vs. model; ## *p* < 0.01 vs. model.

Compared with control group, western diet in model group resulted in significant increase of serous IL-1β, IL-6 and TNF-α levels. Treatment with compound K (3 and 9 mg/kg) and GW3965 significantly reduced the inflammatory cytokine levels above (Figure 2A). Atorvastatin significantly reduced the levels of IL-1β and TNF-α, but not IL-6.

No significant difference occurred in total cholesterol (TC), low density lipoprotein (LDL) cholesterol, very low density lipoprotein cholesterol (VLDL) and triglycerides (TG) levels between model and either of compound K groups. However, significant increase in high density lipoprotein (HDL) cholesterol level was found in compound K (3 and 9 mg/kg) groups compared with model group (Figure 2B).

### 2.3. Compound K Reduces Inflammasome Activity in Atherosclerotic Lesion

**Figure 3:**
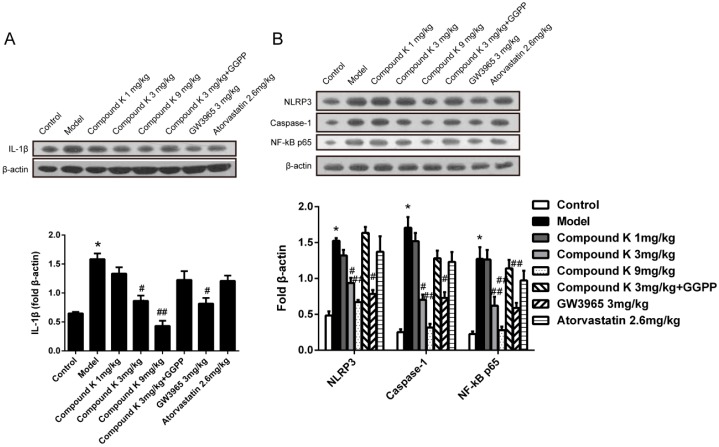
Effects of compound K on inflammasome activity in apoE^−/−^ mice aorta. The level of cleaved-IL-1β in mice aorta was detected by immunoblotting. Treatments of compound K (3, 9 mg/kg) significantly attenuated the increase of cleaved-IL-1β (**A**) expression. To investigate the probably mechanism, the levels of NLRP3, caspase-1 and nuclear NF-κB p65 (**B**) were detected by western blotting, and normalized to β-actin. Data are presented as mean ± SEM (*n* = 6) and analysed by ANOVA with Dunnett’s post-hoc analysis. * *p* < 0.05 vs. control; # *p* < 0.05 vs. model; ## *p* < 0.01 vs. model.

Because of the important role of inflammasome activity on the formation of atherosclerotic lesion, we examined the protein level of cleaved IL-1β, the output of inflammasome activation, in aortic sinus. We found that the treatment with compound K significantly decreased the level of cleaved IL-1β in a dose-dependent manner (Figure 3A). Treatment with compound K (3 and 9 mg/kg) or GW3965 significantly decreased the levels of NLRP3, caspase-1 and NF-κB p65 (Figure 3B). No significant difference occurred in NLRP3, caspase-1 and NF-κB p65 levels between model and atorvastatin groups.

### 2.4. Compound K Promotes the Expression of Reverse Cholesterol Transport (RCT) Related Proteins

**Figure 4:**
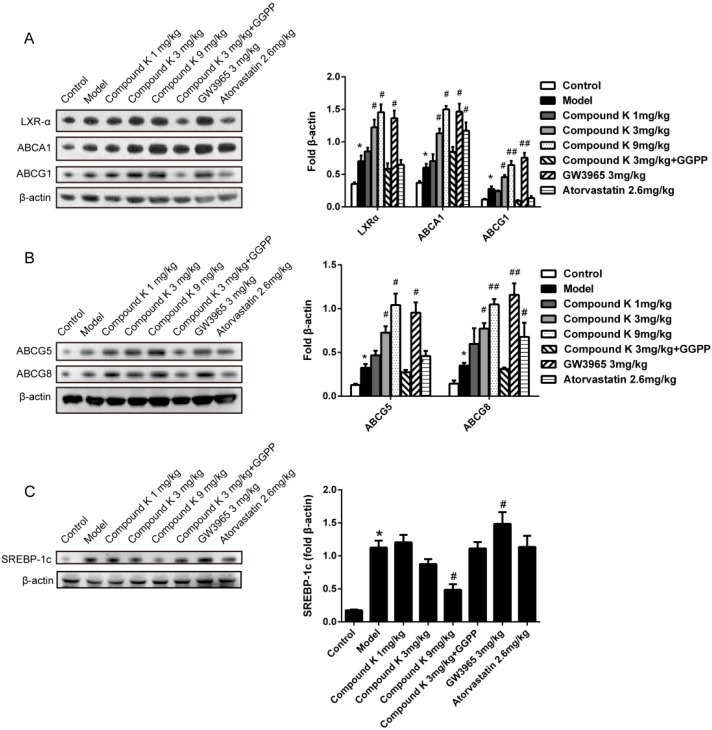
Compound K promotes the expression of reverse cholesterol transport (RCT) related proteins. Levels of LXRα, ABCA1 and ABCG1 in aorta (**A**); ABCG5 and ABCG8 in intestine (**B**); and SREBP-1c (**C**) in liver were detected by immunoblotting and normalized to β-actin. Different from GW3965, treatment of compound K could significantly reduce the expression of SREBP-1c. Data are presented as mean ± SEM (*n* = 6) and analysed by ANOVA with Dunnett’s post-hoc analysis. * *p* < 0.05 vs. control; # *p* < 0.05 vs. model; ## *p* < 0.01 vs. model.

We measured the expression of ABCA1, ABCG1 and LXRα in aorta, and ABCG5 and ABCG8 in intestine by immunoblotting. All of the factors were up-regulated in model group as compared to control group, indicating that the RCT system could be activated by western diet. Treatment with compound K, as well as GW3965, resulted in significant increase of above factors in a dose-dependent manner, compared with model group (Figure 4A,B). Atorvastatin only increased the expressions of ABCA1 and ABCG8. The expression of SREBP-1c in liver was significantly reduced by treatment with compound K, compared with model group (Figure 4C).

### 2.5. Compound K Inhibits the Formation of Foam Cell and the Activity of Inflammasome in Macrophages

**Figure 5:**
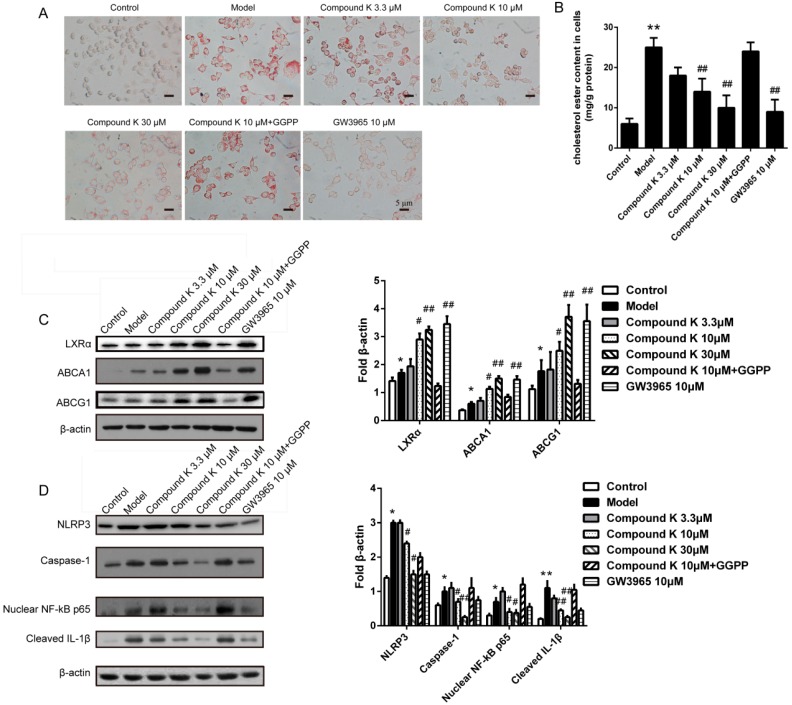
Effects of compound K on formation of foam cells and activity of inflammasome. Foam cells derived from macrophages treated by different concentration compound K were stained by Oil-red O. The visible positive stained lipid drops were detected under microscope (**A**); Concentrations of cholesteryl ester in foam cells were detected (**B**); Levels of LXRα, ABCA1 and ABCG1 in foam cells were detected by immunoblotting and normalized to β-actin (**C**); The level of cleaved-IL-1β in cholesterol crystal (1 mg/mL) stimulated macrophages was detected by immunoblotting (**D**); To investigate the probably mechanism, the levels of NLRP3, caspase-1 and nuclear NF-κB p65 were detected by western blot, and normalized to β-actin (**D**). Data are presented as mean ± SEM (*n* = 6). * *p* < 0.05 vs. control; * *p* < 0.01 vs. control; # *p* < 0.05 vs. model; ## *p* < 0.01 vs. model.

Treatment with compound K (10 and 30 μM) caused a visible decrease in lipid drops deposition as compared to model group in foam cells (Figure 5A), consistent with the result of the measurement of cellular cholesteryl ester. Compared to model group, administration of 10 μM and 30 μM compound K decreased the cholesteryl ester content by 46.21% and 60.24%, respectively (Figure 5B). Then we examined the expression of ABCA1, ABCG1 and LXRα, the major factors in reverse cholesterol transport promotion of macrophage. Compared with model group, dose-dependent changes of ABCA1, ABCG1 and LXRα were demonstrated by the treatments with compound K (Figure 5C).

Furthermore, we investigated the effect of compound K on inflammasome activity in macrophages stimulated by cholesterol crystal. Treatments with compound K (10 and 30 μM) significantly reduced the levels of cleaved-IL-1β, NLRP3, caspase-1 and nuclear NF-κB p65 as compared to model group (Figure 5D).

## 3. Discussion

Atherosclerosis, characterized by the formation of atherosclerotic plaques in the intima of arteries, can cause life-threatening coronary artery disease, carotid artery disease, stroke, and peripheral vascular disease. Commonly practiced pharmacologic therapies of atherosclerosis, including statins and fibrates, are targeted for down-regulation of serous cholesterol or lipid level separately. Nevertheless, the residual risk for major cardiac events remains high for patients receiving LDL lowering therapies [11]. Hence, there is a need for further development of effective therapeutic approaches.

Our previous studies on *Panax notoginseng* saponins demonstrated a significant attenuation of the formation of atherosclerotic plaques by the modulation of serum cholesterol and reduction of inflammatory cytokines [2]. However, the active monomer and the molecular mechanism of *Panax notoginseng* saponins are still unknown. Compound K is a metabolite of Rb1, one of the major components of *Panax notoginseng* saponins. Therefore, we wondered if compound K could play a therapeutic role in atherosclerosis, besides the potential anticancer activity [4,5,6,12]. In addition, synthetic LXRs agonist, GW3965 and lipid-lowering agent, atorvastatin were used as positive control. Firstly, our results indicated that compound K did not induce mortality or impacts on body weight at the doses used in the present study in vivo (Figure S1), consistent with Gao’s study [13].

We have shown here that compound K inhibits the development of atherosclerotic lesions in apoE^−/−^ mice. Reductions in lesion area of approximately 55% were observed in animals treated with compound K, consistent with the change of cholesterol content in aorta. In addition, there was no difference in atherosclerosis lesion area among compound K, GW3965 and atorvastatin groups. The reduction in atherosclerosis caused by compound K treatment was accompanied by the increase in HDL, but no significant change in total cholesterol or LDL, VLDL. The effects of compound K on lipid profiles are likely to involve some reverse cholesterol transport (RCT) related proteins, including ABCA1, ABCG1, ABCG5 and ABCG8. The results above indicated that the athero-protective role of compound K may be associated with the promotion of RCT. Although it is likely that the alterations in serous lipid profiles, such as an increase in HDL [14], may contribute to the anti-atherogenetic effects of compound K, the magnitude of the change does not sufficiently explain the 55% reduction in lesions. Since it is well known that inflammation reaction contributes to the atherogenesis [15], our observations suggest that the effects of compound K on inflammatory reaction contribute to its beneficial effects. Consistent with this hypothesis, we have shown that compound K attenuates the increase of inflammatory cytokines levels in plasma, consistent with previous study [16]. Besides the systemic inflammation, the NLRP3 inflammasome activity in atherosclerotic aortas showed a substantial reduction in response to compound K. The result is consistent with the data showing that compound K can attenuate the inflammasome activation in macrophages pre-stimulated with cholesterol crystal in vitro. Studies have shown that inflammasome activated by cholesterol crystal [17] and mtDNA damage [18] plays a critical role in early stage of atherogenesis via activation of caspase-1 and promotion of pro-inflammatory cytokines IL-1β/IL-18. Although there is no report about the role of compound K in inflammasome activity, studies have shown that compound K can inhibit inflammatory reaction through toll-like receptor and NF-κB signaling pathways [19]. In the present study, besides proving the role of compound K on inflammasome activity, our results indicated that these anti-inflammatory effects of compound K may be associated with LXRα activation. Our results above indicated that besides the effects on lipoprotein metabolism, compound K can reduce inflammation caused by inflammasome activation in atherosclerotic aorta. Furthermore, our results showed that RCT promotion and anti-inflammation effects of compound K could be reversed by LXRα inhibitor, GGPP, which indicates that LXRα pathway might play a major role in athero-protection of compound K.

Since our previous studies have indicated that the athero-protective effects of *Panax notoginseng* saponins contribute to LXRα activation, it is probable, based on current observations, that compound K is mechanistically associated with LXRα. The characterization of LXRα as regulator of RCT has generated widespread interest in the development of LXRα ligands as therapeutics [20]. In addition, studies have shown that tGW3965, as well as other synthetic LXR agonists, could inhibit the development of aortic lesions in animal models [21]. Studies show that the activation of LXRα, one of the LXRs, attenuates the atherosclerosis formation by an increase in expression of cholesterol efflux transporters ABCA1 and ABCG1 in macrophages [21], and excretion transporters ABCG5 and ABCG8 in enterocytes [22]. However, unfortunately, synthetic agonists of LXRα, such as T090317 and GW3965, can result in the increase of SREBP-1c expression, leading to the elevation of plasma triglycerides [23] and liver steatosis [24]. These serious side effects presented a major obstacle in the development of synthetic LXRα agonists as anti-atherosclerosis drugs. In addition, 27-hydroxycholesterol, another LXRα agonist, even promotes atherosclerosis via proinflammatory processes [25]. In the present study, we have shown that compound K up-regulates the expression of the downstream target proteins of LXRα and compound K selectively activates LXRα reporter plasmid (Figure S2) in vitro. Yang’s study suggested compound K as a novel agonist of glucocorticoid receptor [7], which can up-regulate the expression of ABCA1. However, our results that GGPP can reverse most effects of compound K on ABCA1 indicated that LXRα-dependent mechanism played a major role in this experiment. Furthermore, study showed the roles of compound K on vascular smooth muscle cell proliferation and migration through cell cycle related-proteins mechanism [26]. These effects also may contribute to the anti-atherosclerosis roles of compound K. In addition, the possible relationship between LXRα pathway and vascular smooth muscle cell proliferation needs to be explored in the future. Besides the pharmacodynamic roles, interestingly, unlike the LXRα agonists above, compound K did not induce the triglycerides increase and hepatic steatosis. Instead, the liver steatosis in mice was attenuated by compound K, the probably mechanism may be associated with the reduction of SREBP-1c expression [27].

In summary, our results show that compound K, an active monomer of *Panax notoginseng* saponins, attenuates the atherosclerosis formation in apoE^−/−^ mice, through RCT promotion and inflammasome inhibition in association with LXRα activation. Moreover, compound K does not exhibit the common serious side effect, lipidogenetic promotion, of synthetic LXRα agonists. Therefore, compound K might have the potential to be an effective drug for the prevention and treatment of atherosclerosis.

## 4. Materials and Methods

### 4.1. Mice and Treatments

Ninety-six male C57BL/6 apoE^−/−^ mice aged 10 weeks (22–25 g) were purchased from Peking University Health Science Center (Beijing, China). All animals underwent a common 1 week acclimatization period and were maintained on a 12 h light-dark cycle at the local animal facility at the Animal Center of the Third Military Medical University, under specific pathogen-free conditions. The body weights of mice were monitored once a week.

The mice were randomly divided into 8 groups (*n* = 6). All animals were provided with unlimited access to water and western diet (basic feed containing 0.5% cholesterol and 5% lard) except the control group, which were provided basic feed. Drugs were administered by intra-peritoneal injection once a day for 8 weeks. Compound K (E-0120, Tauto Biotech Co., Ltd., Shanghai, China) was suspended in normal saline buffer and the doses were 1, 3 and 9 mg/kg respectively. As positive control, mice were treated with GW3965 (3 mg/kg, Sigma-Aldrich, St. Louis, MO, USA) or atorvastatin (2.6 mg/kg, Sigma-Aldrich, St. Louis, MO, USA) respectively. GGPP group was administrated with compound K (3 mg/kg) plus 9 mg/kg GGPP (Sigma-Aldrich, St. Louis, MO, USA). Model group was treated with normal saline.

Animals were fasted overnight and euthanized, and blood was collected from the abdominal vena cava. Then mice were sacrificed by CO_2_ inhalation in accordance to the AVMA guidelines for the euthanasia of animals, 2013 edition. Tissue samples, including aorta, liver and intestine, were dissected for the sequential experiments.

All procedures were performed conform the NIH guidelines (Guide for the care and use of laboratory animals) and the Animal Management Rules of the Ministry of Health of the People’s Republic of China (No. 55, 2001), the protocol was previously approved by the Institutional Ethics Committee for Use of Animals at Third Military Medical University (Chonqing, China).

### 4.2. Histology and Image Analysis

For en face analysis, aorta from heart to iliac bifurcation aorta was fixed by perfusion with 10% formalin for 50 min. Then aorta was opened longitudinally, and the entire aorta was stained with Oil-Red O for 15 min. Images were captured for the following analysis.

The upper portion of the heart and proximal aorta were obtained and paraffin-embedded. Serial 10 μm-thick sections of aorta, beginning at the aortic root, were collected for a distance of 600 μm. Sections were stained with Oil Red O and hematoxylin. The lipid-staining areas on 24 sections in each group were determined in a blinded fashion by Nikon Eclipse 90i light microscope (Nikon Instruments, Melville, NY, USA). The mean value of lesion area of aortic wall per section was then calculated.

### 4.3. Determination of Serous Lipid and Cytokines

Mice were fasted overnight and euthanized. Blood was collected by removalling eyeballs and centrifuged by 1000× *g* for 10 min. Then supernatants were collected as the plasma samples for further detection. The levels of serous lipid including total cholesterol (TC), triglyceride (TG), high-density lipoprotein (HDL) cholesterol and low-density lipoprotein (LDL) cholesterol were detected by an AU-2700 automatic biochemical analyzer (Olympus, Tokyo, Japan). The detection kits were purchased from Kang Taike Medical Co., Ltd. (Beijing, China), while the standard serous sample was supplied by Randox Laboratories Ltd. (Northern Ireland, UK).

The serous levels of tumor necrosis factor-α (TNF-α), interleukin-1β (IL-1β) and interleukin-6 (IL-6) were determined by Mcytomag-70K-3, Mouse Cytokine/Chemokine Magnetic Bead Panel (Merck Millipore Co., Ltd., Billerica, MA, USA) according to the manufacturer’s protocol.

### 4.4. Cell Culture and Treatments

Peritoneal macrophages were obtained from apoE^−/−^ mice by Zeini’s method [28]. Briefly, mice were injected i.p. with 3 mL Dulbecco’s Modified Eagle Medium (DMEM) (Hyclone, Invitrogen Corporation, Grand Island, NY, USA). After 30 min, animals were sacrificed by CO_2_ inhalation as described above. The fluid of peritoneal cavity was carefully collected and kept at 4 °C. After centrifugation at 200× *g* for 10 min at 4 °C, the pellet was washed twice with ice-cold PBS. Cells were adjusted to 1 × 10^5^/mL in DMEM containing 10% (*v*/*v*) fetal bovine serum (Hyclone), 100 U/mL of penicillin and 100 μg/mL of streptomycin, and incubated at 37 °C in a humidified atmosphere of 5% CO_2_. Non-adherent cells were removed 2 h after seeding by extensive washing with medium.

The macrophages were incubated with 100 μg/mL oxidized low density lipoprotein (ox-LDL, MDA 30 μM, Institute of Basic Medicine, Peking Union Medical College, Beijing, China) in 6-well plates for 24 h. Then cells were administrated with ox-LDL free medium containing CD36 blocking antibody (Santa Cruz Biotechnology Inc., Santa Cruz, CA, USA, 1:1000) and compound K (0 μM, 3.3 μM, 10 μM, 30 μM) or GW3965 (3.3 μM) or compound K (10 μM) plus 10 mM GGPP for another 24 h. Cells of control group were incubated over the same time period in culture media without any treatment.

Cholesterol crystal was prepared by Rajamäki’s method [29]. Briefly, cholesterol (Shanghai Sangon Biotech Co., Ltd., Shanghai, China) was dissolved in 95% ethanol (12.5 g/L), then heated to 60 °C, filtered while warm, and placed at room temperature to allow crystallization to form (1–5 mm, bright, rhomboid and relatively large). The crystal was collected and autoclaved, grinded to yield a size range of 1–10 μm, and stored at −20 °C until use.

For studying the effects of compound K on inflammasome activity in macrophages, the cells were cultured with cholesterol crystals (1 mg/mL) for 24 h, and then were administered as described above. Then cells were collected for future measurements.

For reporter gene experiment, HEK293T cells were adjusted to 5 × 10^5^/mL in DMEM containing 10% (*v*/*v*) fetal bovine serum (Hyclone), and incubated at 37 °C in a humidified atmosphere of 5% CO_2_. pCMX-hLXR-α or pCMV-hLXR-β, hLXREx3TK-Luc (reporter plasmid), and pSV-β-galactosidase (transfection efficiency control) were transfected into HEK293T cells with Lipofectamine 2000 Transfection Reagent (Life Technologies, Grand Island, NY, USA). Transfected cells were cultured for 48 h prior to further treatments. After washed with PBS, cells were treated with different concentrations of compound K (3.3, 10 μM). GW3965 (3.3, 10 μM) was used as positive control. Then cells were lysed, and assayed for luciferase and β-galactosidase activities.

### 4.5. Immunoblotting

Freshly obtained tissues were weighed, and 1 mL of T-PER protein extraction reagent (Pierce Labs, Rockford, IL, USA) and protease inhibitor cocktail (Sigma-Aldrich-Aldrich, St. Louis, MO, USA) was added for every 100 mg tissue. The tissue was then homogenized with a Bullet Blender Blue (Next Advance, Averil Park, NY, USA) at setting 7 for 8 min at 4 °C. After homogenization, the suspension was centrifuged at 10,000× *g* for 5 min at 4 °C, and supernatant was collected.

Cells in six-well plates were extracted with 300 μL/well of protein extraction reagent (T-PER) and protease inhibitor cocktail (Sigma-Aldrich) for 2 h at 4 °C with gentle shaking. The extraction was centrifuged at 10,000× *g* for 5 min and the supernatant was collected. The nuclear protein extracts were selectively isolated with NE-PER Nuclear and Cytoplasmic Extraction Reagents (Pierce Labs, Rockford, IL, USA) using the manufacturer’s protocol.

All of the protein samples were determined using the BCA assay and stored at −80 °C until analyzed. Extracts were resolved by SDS gel electrophoresis on 4%–12% NuPAGE Bis-Tris polyacrylamide gels for 50 min at 150 V. Resolved proteins were electro-blotted onto PVDF membranes (iBlot, Life Technologies, Gaitherburg, MD, USA). Membranes were blocked with 5% nonfat milk in PBS containing 0.1% Tween 20 (PBST) for 30 min. Blots were washed three times in PBST and incubated with target primary antibodies for 16 h at 4 °C specifically. After three times wash, blots were incubated with species-specific horseradish peroxidase (HRP)–conjugated secondary antibodies (1:2000 in PBST) for 1 h at room temperature. Blots were washed three times in PBST and then were detected using enhanced chemiluminescence (ECL, GE Healthcare). ECL images were collected using a Chemidoc-XRS image system (Bio-Rad, Hercules, CA, USA), bands quantified using Quantity One software (Bio-Rad, Hercules, CA, USA), normalized to β-actin expression.

As a loading control, extracts were resolved on 4%–12% NuPAGE gels, blotted to PVDF, and β-actin detected using HRP-conjugated anti-β-actin antibody (1:2000, Kangchen Inc., Kangchen, Shanghai, China) with ECL detection as described above.

The following antibodies were used: anti-LXRα (Santa Cruz Biotechnology, Dallas, TX, USA, sc-1202, 1:500), anti-ABCA1 (Santa Cruz, sc-20794, 1:500), anti-ABCG1 (Santa Cruz, sc-20795, 1:500), anti-ABCG5 (Santa Cruz, sc-25796, 1:500), anti-ABCG8 (Santa Cruz, sc-30111, 1:500), anti-NLRP3 (Santa Cruz, sc-66846, 1:200), anti-Caspase-1 (Santa Cruz, sc-56036, 1:200), anti-cleaved IL-1β (Santa Cruz, sc-23460, 1:500), anti-NF-κB p65 (Santa Cruz, sc-398442, 1:500), and anti-SREBP1 (Santa Cruz, sc-366, 1:500).

### 4.6. Analysis of Cholesterol Ester Content of Aorta and Foam Cells

Foam cells stained with Oil-red O were observed under a Nikon Eclipse 90i microscope (Nikon, Tokyo, Japan). Extraction and analysis of cellular cholesterol ester (CE) content were described in a former study [4]. Briefly, cholesterol samples of aorta and foam cells (1 well) were extracted with a chloroform/methanol (2:1; *v*/*v*) mixture, and detected with total cholesterol (TC) and free cholesterol (FC) content measurement reagent (Rongshen Inc., Shanghai, China), and CE content was obtained by calculation (CE = TC − FC). The sediments were collected for the measurement of protein concentration.

### 4.7. Statistical Analysis

All data in this study are expressed as the mean ± SEM. Statistical analysis was performed using SPSS 13.0 software (SPSS Inc., Chicago, IL, USA). Comparisons between groups were made using one-way ANOVA analysis with post-hoc least significant difference (LSD) test. Values of *p* < 0.05 were considered to be statistically significant.

## Supplementary Materials

Supplementary materials can be found at http://www.mdpi.com/1422-0067/17/7/1054/s1.

## Abbreviations

ABC	ATP-binding cassette transporter
GGPP	Geranylgeranyl pyrophosphate
HDL	high density lipoprotein
IL	interleukin
LDL	low density lipoprotein
LXRα	liver X receptor α
NF-κB	nuclear factor kappa B
NLRP3	NOD-like receptor protein-3
RCT	reverse cholesterol transport
SREBP	sterol regulatory element-binding protein
VLDL	very low density lipoprotein
