# Supplementary material for: Compound K Attenuates the Development of Atherosclerosis in ApoE−/− Mice via LXRα Activation
Source: Int J Mol Sci. 2016 Jul 8;17(7):1054. doi: 10.3390/ijms17071054 (PMC4964430; doi:10.3390/ijms17071054)
Supplement: Supplementary file 1 [file ijms-17-01054-s001.pdf]

Supplementary Materials: Compound K Attenuates the Development of Atherosclerosis in ApoE$^{-/-}$ Mice via LXRα Activation

Li Zhou, Yu Zheng, Zhuoying Li, Lingxia Bao, Yin Dou, Yuan Tang, Jianxiang Zhang, Jianzhi Zhou, Ya Liu, Yi Jia and Xiaohui Li

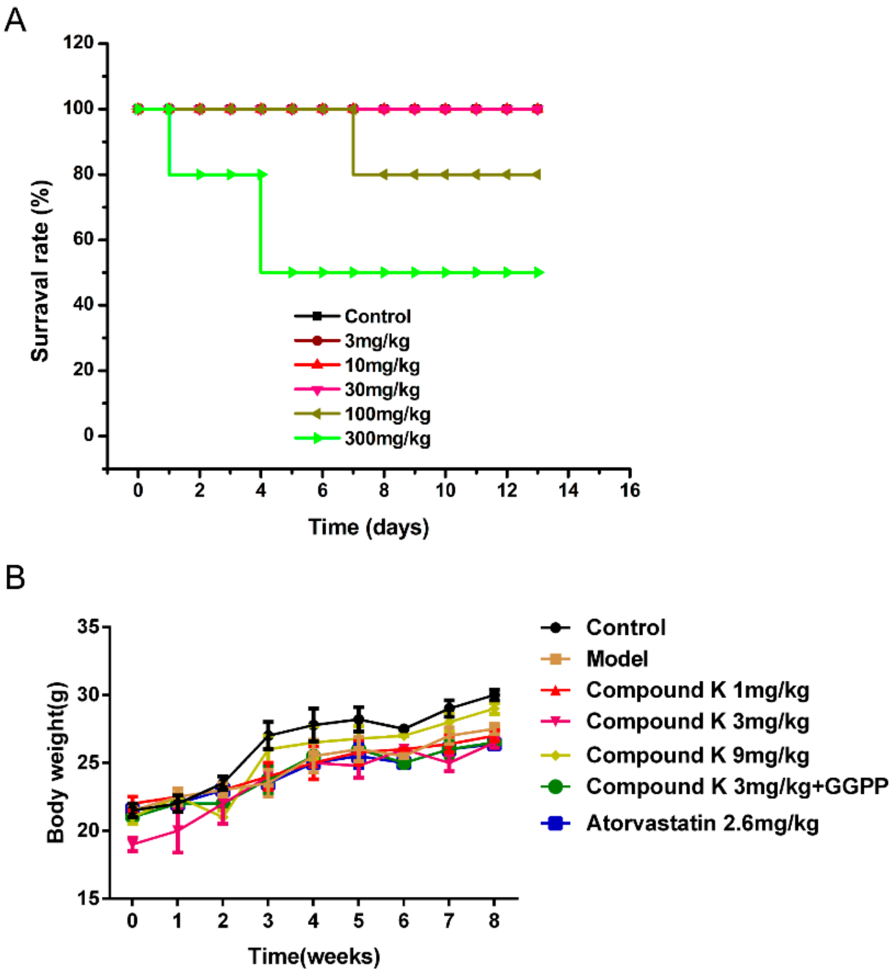

Figure S1. Potential acute toxicity of Compound K in vivo. Compound K of different concentration was administered to mice i.p. once. Survival ratio was recorded after 14 days. There was no mouse got dead until the compound K dose reached 100 mg/kg (**A**); During the in vivo experiments, animals were weighed every week. There was no significant alteration in the body weight (**B**). Data of weight are presented as mean ± SEM (n = 6), and analysed by ANOVA with Dunnett's post-hoc analysis.

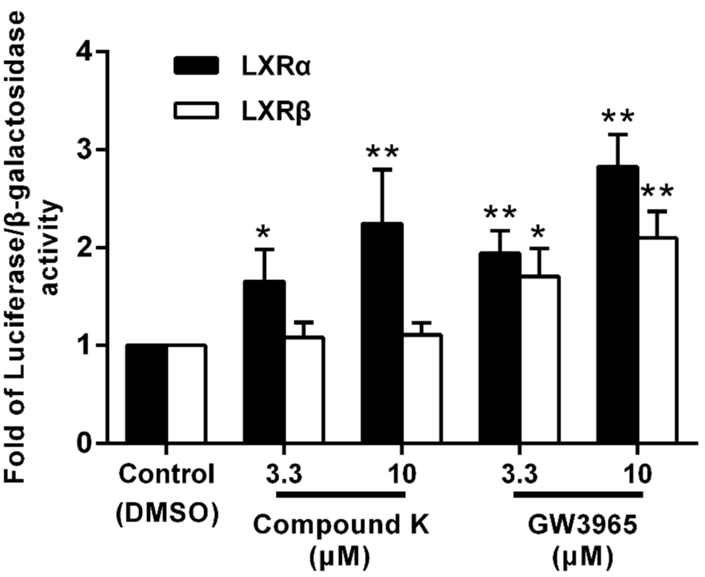

Figure S2. Compound K selectively actives LXR-α in HEK293T cell line. The HEK293T cells were transfected with hLXREx3TK-Luc as a reporter plasmid, pCMX-hLXR-α or pCMV-hLXR-β as expression vectors, and pSV-β-galactosidase was used to normalise the transfection efficiencies. Cells were treated with increasing concentrations of compound K (3.3, 10 μM). GW3965 (3.3, 10 μM) was used as positive control. After incubation, the cells were lysed, and assayed for luciferase and β-galactosidase activities. The results are expressed as relative luciferase activity (fold difference compared to negative control). Data are presented as mean ± SEM (n = 3, each in duplicate). * $p < 0.05$ vs. control, ** $p < 0.01$ vs. control.
